# Adaptive enhancement of shoulder x-ray images using tissue attenuation and type-II fuzzy sets

**DOI:** 10.1371/journal.pone.0316585

**Published:** 2025-02-06

**Authors:** Qifeng Liu, Yong Han, Lu Shen, Jialei Du, Marzia Hoque Tania

**Affiliations:** 1 Centre for Big Data Research in Health, University of New South Wales, Sydney, Australia; 2 School of Software Engineering, Xi’an Jiaotong University, Xi’an, China; 3 International Digital Economy College, Minjiang University, Fuzhou, China; 4 Business School, University of New South Wales, Sydney, Australia; Islamia University of Bahawalpur: The Islamia University of Bahawalpur Pakistan, PAKISTAN

## Abstract

Shoulder X-ray images typically have low contrast and high noise levels, making it challenging to distinguish and identify subtle anatomical structures. While existing image enhancement techniques are effective in improving contrast, they often overlook the enhancement of sharpness, especially when amplifying blurring and noise. These techniques may improve detail contrast but fail to maintain overall image clarity and the distinction between the target and the background. To address these issues, we propose a novel image enhancement method aimed at simultaneously improving both the contrast and sharpness of shoulder X-ray images. The method integrates automatic tissue attenuation techniques, which enhance the image contrast by removing non-essential tissue components while preserving important tissues and bones. Additionally, we apply an improved Type-II fuzzy set algorithm to further optimize image sharpness. By simultaneously enhancing contrast and sharpness, the method significantly improves image quality and detail distinguishability. When tested on certain images from the MURA dataset, the proposed method achieved the best or second-best results, outperforming five no-reference image quality assessment metrics. In comparative studies, the method demonstrated significant performance advantages over 10 contemporary X-ray image enhancement algorithms and was validated through ablation experiments to confirm the effectiveness of each module.

## Introduction

Shoulder X-ray imaging plays a crucial role in the healthcare industry, widely used for disease diagnosis, prevention, and in medical research and education, significantly enhancing the accuracy of disease diagnoses and improving patient health outcomes. However, shoulder radiographs often come with several flaws, such as noise [1], low contrast [2], blurriness [3], and a limited dynamic range [4]. Noise may mask essential details critical for early disease detection. Low contrast complicates the differentiation of tissue types, potentially leading to misinterpretation [5]. Blurriness reduces the sharpness necessary for identifying organ boundaries or lesions, and a restricted dynamic range can cause loss of detail in both overly dark and bright areas [6]. These quality issues collectively risk diagnostic accuracy, potentially leading to delayed or inappropriate treatment decisions, thereby affecting patient outcomes [7]. Image enhancement techniques that reduce noise, blur, and artifacts can assist doctors in accurately identifying and interpreting details within X-ray images, thereby increasing diagnostic precision.

Methods based on tissue attenuation [8] have been extensively applied for X-ray image enhancement, reducing nonessential information and enhancing the contrast of key tissues, organs, and bones. This enables doctors to quickly recognize and analyze structures critical for diagnosis. Nevertheless, these methods often overlook the characteristics of low sharpness and high noise levels inherent in X-ray images. Given the low sharpness, crucial details may be lost, and the presence of significant noise levels can interfere with the performance of tissue attenuation-based enhancement algorithms, leading to suboptimal enhancement results. Overcoming these challenges may require the integration of multiple image processing techniques to effectively enhance the images.

Recently, significant advancements have been made in traditional and deep learning-based methods for X-ray image enhancement. For traditional enhancement methods, Koonsanit et al. [9] proposed an adaptive histogram method to enhance image detail, texture, and local contrast. Veluchamy et al. [10] proposed an adaptive gamma correction with weighted histogram distribution method to improve contrast while preserving natural color and richer details. Fu et al. [11] explicated an X-ray image enhancement algorithm based on an improved Retinex-Net [12], effectively enhanced visual clarity, contrast, and detail while reducing noise in X-ray images. For deep learning methods, Ma et al. [13] proposed a bi-directional GAN [14] with local structure and illumination constraints, improving medical image quality for various clinical tasks. Madmad et al. [15] elucidated a method for enhancing X-ray images by separating them into local textures and smooth parts using convolutional neural networks trained with synthetic data, resulting in improved visualization. Zhong et al. [16] proposed a multi-scale attention generative adversarial network for enhancing medical images by addressing issues of illumination distribution, texture details, and artifact noise, achieving superior enhancement results. However, these advancements often overlook the substantial improvements that can be achieved by incorporating sharpness enhancement techniques.

**Fig 1:**
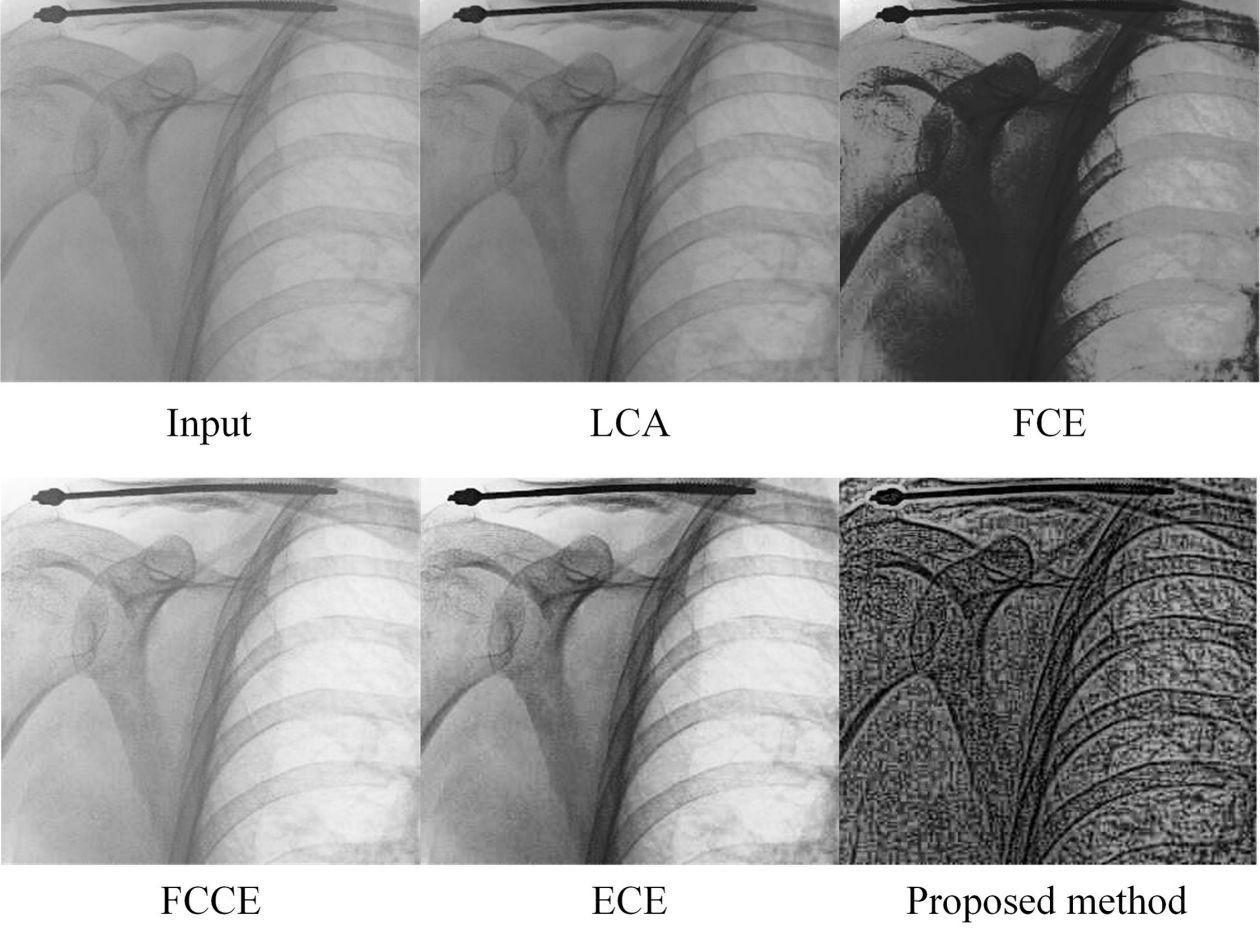
Our proposed enhancement technique exhibits enhanced performance in elevating X-ray image quality relative to established methodologies, including LCA [47], FCE [44], FCCE [43], and ECE [41].

We propose a dual-image enhancement strategy that simultaneously improves image contrast and sharpness, significantly enhancing overall image quality. First, an automatic tissue attenuation technique preserves essential tissues, organs, and bones while removing unnecessary components, thus enhancing contrast. Then, a sharpness enhancement algorithm based on type-II fuzzy sets is applied to improve image sharpness. The combination of these strategies leads to a significant improvement in X-ray image quality. Notably, our method outperforms many traditional X-ray image enhancement techniques, as shown in Fig 1, where it clearly displays skeletal structures and key tissues. In summary, our research makes four key contributions to the field of shoulder X-ray imaging:

- We proposed a tissue attenuation-based contrast enhancement technique that removes non-essential tissue components, enhancing image contrast by preserving important tissues and bones.
- We proposed a type-II fuzzy set-based sharpness enhancement algorithm, significantly improving shoulder X-ray image sharpness by emphasizing subtle, diagnostic-critical details.
- Our approach combines sharpness and contrast enhancements, significantly boosting image quality on both fronts.
- We present the validation of the MURA dataset in the field of shoulder X-ray image enhancement. Our proposed method was evaluated using five no-reference image quality assessment metrics and achieved optimal or sub-optimal performance by comparing 10 image enhancements.

This study presents a comprehensive method aimed at addressing issues of low contrast and poor sharpness in shoulder X-ray images. The method combines multiple processing strategies to enhance both contrast and sharpness, thereby improving support for medical diagnosis. The paper begins by introducing the importance of shoulder X-ray imaging and discussing the challenges of image enhancement under low sharpness conditions. It then reviews traditional methods and deep learning techniques used in X-ray image processing, providing a theoretical foundation for the proposed approach. The methods section details the application of tissue attenuation techniques and type-II fuzzy sets in image enhancement. The experiments section explains qualitative and quantitative experiments validate the effectiveness of the method, and an ablation study further confirms the contribution of each module. The discussions section summarizes the current research and addresses its limitations. Finally, the conclusion summarizes the research findings, emphasizes the improvement in medical imaging quality, and looks ahead to future research directions. A complete list of abbreviations used in the paper is provided in Table A1 in S1 Appendix.

## Related work

Recent advancements in medical imaging, particularly in X-ray image enhancement, have been driven by the fusion of traditional image processing techniques and deep learning algorithms. Traditional methods, including histogram equalization, retinex theory, gamma correction, and spatial filtering, have been fundamental in adjusting image pixel values to improve contrast and clarity. This has enabled medical practitioners to make more accurate diagnoses by enhancing image visualization and highlighting critical details. On the other hand, deep learning approaches like convolutional neural networks [17] and generative adversarial networks [18] have revolutionized the field by automatically extracting and utilizing complex patterns from large datasets to produce clearer and more contrasted images. In summary, image enhancement techniques are crucial for downstream tasks in computer vision. Through effective image enhancement and preprocessing, low-quality medical images can be improved, helping professionals make critical decisions based on the images [19].

### Traditional medical image enhancement methods

In the realm of medical imaging, particularly X-ray image enhancement, traditional enhancement methods have played an important role in addressing various challenges associated with image quality. A range of techniques have been developed to tackle issues like excessive brightness, foggy appearance due to dense tissue, non-uniform luminance, low contrast, and the preservation of natural colors and details, such as Huang et al. [8] addressed images with excessive brightness and foggy appearance due to dense tissue by proposing a decomposition of X-ray images into tissue and detail components. They enhanced visual contrast through adaptive tissue attenuation and dynamic range stretching and implemented an ensemble framework to fuse images from dark and bright regions. Their results effectively highlighted organs, bone structures, and significant small details. For DICOM [20] images, which often suffer from non-uniform luminance and low contrast, few methods are suitable, Zhao et al. [21] introduced a luminance-level modulation and gradient modulation technique, compressing the luminance range to improve visual quality and enhance image details to increase contrast while avoiding staircase artifacts and outperforming current leading methods. However, its reliance on luminance and gradient modulation could lead to challenges in preserving fine details in images with highly variable intensity distributions, requiring careful tuning for optimal performance in differentclinical scenarios. Tao et al. [22] designed a retinex-based image enhancement framework that using region covariance filter at different scales to estimate the illumination and adopted contrast-limited adaptive histogram equalization, non-local means filter, and guided filter to enhance the contrast, eliminate the noise, and increase the details of original images respectively. To address the issue of insufficient contrast in cardiac MRI videos, Jabbar et al. [23] proposed a method based on fuzzy image technology. Through steps such as fuzzification, membership function adjustment, and defuzzification, the method effectively enhances the video contrast, demonstrating improved performance compared to other approaches.

Recent traditional image enhancement methods for X-rays have evolved to better address the multifaceted challenges of medical imaging. Yadav et al. [24] utilized an entropy curve and homomorphic filtering to improve image contrast and detail without amplifying noise and artifacts, mitigating the typical under or over enhancement in dark or bright areas. Nevertheless, the complexity of the filtering process may present challenges for real-time application in clinical environments. Jabbar et al. [25] proposed a local fuzzy inference technique to enhance the contrast and visual quality of musculoskeletal ultrasound images, demonstrating improved performance and efficiency, with potential applications as a preprocessing step for tasks like image segmentation and 3D reconstruction. To address noise, improper exposure, and obscured details in digital radiography images, Liu et al. [26] proposed a method using wavelet multiscale decomposition with Shannon–Cosine wavelets. This approach segmented images across frequencies, enhancing diagnostic information while suppressing noise through region-specific attenuation coefficients, effectively improving image clarity and robustness. To deal with low contrast in medical images, Khan et al. [27] proposed the Fast Local Laplacian Filter, which selectively enhances low-contrast areas while preserving edges and fine details, improving visual quality and reducing noise levels to aid in accurate diagnosis.

However, image enhancement based on traditional methods faced several challenges. These methods often led to a trade-off between detailed enhancement and noise reduction, failing to uniformly improve image quality across various regions. They relied heavily on manual adjustments, making the process time-consuming and inconsistent. Traditional approaches also struggle with the diverse and complex nature of medical images, which contain a wide range of contrasts and details critical for diagnosis. Their one-size-fits-all strategy frequently overlooked the nuanced differences between tissues and conditions, potentially masking important diagnostic information. This highlighted the need for more advanced, adaptable solutions capable of addressing the unique challenges of X-ray image enhancement. The various traditional methods for X-ray image enhancement discussed in this section are summarized in Table 1.

**Table 1:** Summary of traditional image enhancement method for medical images and their applications.

Authors	Method/Technique	Application
Huang et al. [8]	Adaptive tissue attenuation and dynamic range stretching	Enhancing X-ray images with excessive brightness and foggy appearance
Zhao et al. [21]	Luminance-level modulation and gradient modulation	Improving DICOM images with non-uniform luminance and low contrast
Tao et al. [22]	Retinex-based enhancement with various filters	Enhancing contrast and detail of original medical images
Jabbar et al. [23]	Fuzzy image technique with membership function adjustment	Enhancing video contrast in cardiac MRI
Yadav et al. [24]	Entropy curve and homomorphic filtering	Balancing contrast and noise reduction in X-rays
Jabbar et al. [25]	Local fuzzy inference technique	Enhancing musculoskeletal ultrasound images
Liu et al. [26]	Wavelet multiscale decomposition with Shannon–Cosine wavelets	Improving clarity of digital radiography images
Khan et al. [27]	Fast Local Laplacian Filter	Enhancing low-contrast medical images

### Deep learning medical image enhancement methods

Building on the foundation set by traditional image enhancement techniques, deep learning strategies have introduced a paradigm shift in medical imaging, particularly by enhancing image quality with a focus on detail preservation and overcoming some limitations of traditional methods. Confronting the challenge of limiting paired samples, Yu et al. [28] developed a fuzzy self-guided structure retention generative adversarial network. This network comprises a self-guided structure retention module and an illumination distribution correction module. The network focuses on preserving essential structural information in nerve fibers, while the illumination distribution correction module harmonizes illumination distribution for clearer medical structure visualization. However, the model may encounter challenges with generalization to datasets beyond nerve fiber images, as its architecture is tailored to specific structural features, potentially limiting its broader applicability. The network produced enhanced images with uniform illumination and rich texture. Addressing the quality of fundus images, Wu et al.[29] introduced a semi-supervised GAN with anatomical structure preservation to navigate around the limitations of specific prior knowledge requirements and generalizability issues plaguing existing enhancement methods. Zhong et al. [16] identified that maintaining texture details and preventing boundary artifact noise is a significant limitation of current enhancement techniques. They proposed the multi-scale attention generative adversarial network, which is designed specifically for medical images and performs well with unpaired datasets, demonstrating notable improvements in image analysis and segmentation tasks. Nevertheless, reliance on unpaired datasets, while advantageous, could also introduce variability in outcomes, making consistent performance across different medical imaging modalities more difficult to achieve. Ma et al. [13] presented the novel structure and illumination constrained GAN, categorizing images by quality and applying constraints that balance overall quality enhancement with detail preservation. Furthermore, Qiu et al. [30] developed an image enhancement method combining curvelet transformations, frequency band broadening, and CNNs, focusing on edge detail and noise reduction in medical images. Their approach, involving artifact mitigation and resolution enhancement, resulted in improved diagnostic quality across multiple imaging modalities. While deep learning methods in medical image enhancement boast significant advantages, such as the ability to learn complex patterns and improve image quality autonomously, their drawbacks include being heavily dependent on the availability and quality of datasets and a lack of interpretability. These methods require large, well-annotated datasets to train effectively, which can be a challenge in medical settings due to privacy concerns and the labor-intensive nature of annotation.

Additionally, deep learning models are often described as "black boxes" because their decision-making processes are not easily understandable, raising concerns about reliability and trustworthiness in critical medical applications. Phan et al. [31] introduced a novel two-stage approach for enhancing the quality and privacy of X-ray medical images. The first stage utilizes generative adversarial networks to effectively denoise the images, enhancing visibility of critical anatomical structures by eliminating noise and artifacts. Subsequently, the method integrates number-theoretic transform polynomial multiplication to accelerate the encryption and decryption processes, ensuring privacy protection without relying on Kyber encryption [32]. Madmad et al. [15] presented a new method for improving X-ray images by separating them into local textures and smooth areas using a dual-branch convolutional neural network. The approach focused on distinguishing the detailed textures from the overall smooth shapes in the images. Trained on synthetic data, this CNN effectively decomposed images into their two key components. The technique emphasized enhancing the texture details, resulting in images that outperformed those produced by traditional methods for high dynamic range visualization, such as tone-mapping algorithms.

However, deep learning for medical image enhancement, such as X-ray imaging, faced key challenges, including a reliance on extensive, annotated datasets that were difficult to obtain due to privacy and expertise requirements. The lack of interpretability in black-box models raised trust issues among healthcare professionals. These methods struggled with generalization, high computational demands, security risks, and biases in training data, highlighting the need for ethical and effective deep learning in medical image enhancement. The deep learning-based methods for medical image enhancement covered in this section are outlined in Table 2.

**Table 2:** Summary of deep learning-based image enhancement methods for medical images and their applications.

Authors	Method/Technique	Application
Yu et al. [28]	Fuzzy self-guided structure retention GAN	Preserving nerve fiber structures and harmonizing illumination
Wu et al. [29]	Semi-supervised GAN with anatomical structure preservation	Improving fundus image quality
Zhong et al. [16]	Multi-scale attention GAN	Maintaining texture details in medical images
Ma et al. [13]	Structure and illumination constrained GAN	Balancing quality and detail in medical images
Qiu et al. [30]	Curvelet transforms, frequency band broadening, and CNNs	Enhancing edge detail and reducing noise
Phan et al. [31]	GAN-based denoising with privacy protection	Denoising and protecting X-ray image privacy
Madmad et al. [15]	Dual-branch CNN for local texture enhancement	Enhancing texture for high dynamic range visualization

## Method

In addressing the inherent challenges presented by X-ray imaging, the low contrast and low sharpness which complicate diagnosis, we proposed a two-fold enhancement approach. Firstly, our method leverages tissue attenuation to mitigate the interference of non-critical tissue components that may otherwise overlap with vital organs or tissues, obscuring crucial details and leading to diagnostic ambiguities. By selectively attenuating certain tissue elements, we preserve and accentuate the fine details, thereby enriching the contrast of the image. Concurrently, the sharpness of X-ray images plays a critical role, as it underpins the ability of healthcare professionals to discern subtle yet significant anomalies within these internal structures. To ameliorate issues of sharpness in X-ray images, we incorporated an algorithm rooted in type-II fuzzy set for sharpness enhancement, effectively increasing the perceptibility of the imagery. This comprehensive methodology forms the bedrock of our proposed method, integrating contrast enhancement with sharpness improvement to support more effective diagnostic imaging. This section mainly includes three parts. First, a method to enhance the contrast of low dynamic shoulder X-ray image.

**Fig 2:**
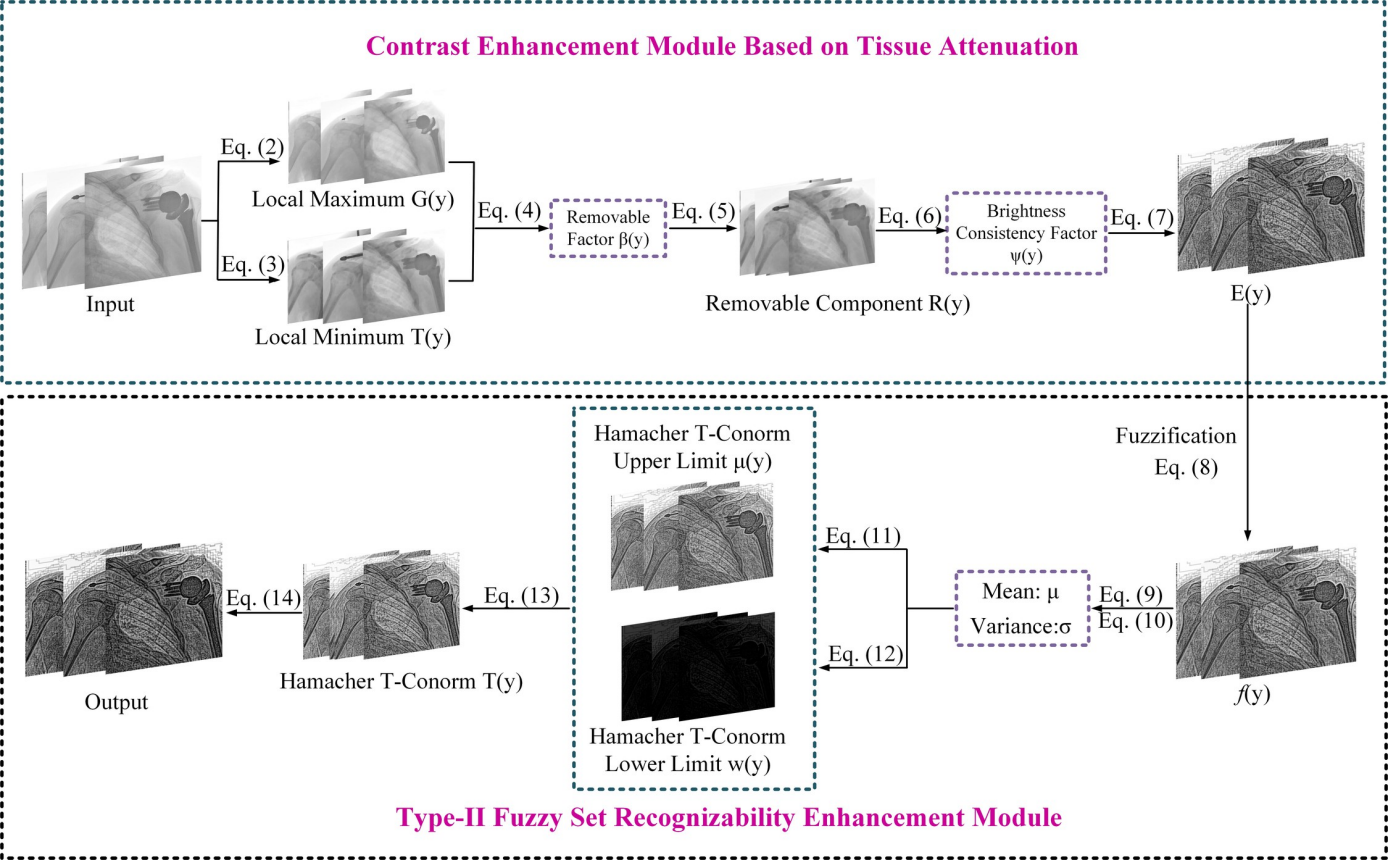
The flowchart of the proposed method outlines a two-stage image enhancement algorithm. The first stage focuses on contrast enhancement. The second stage aims at sharpness enhancement.

**Fig 3:**
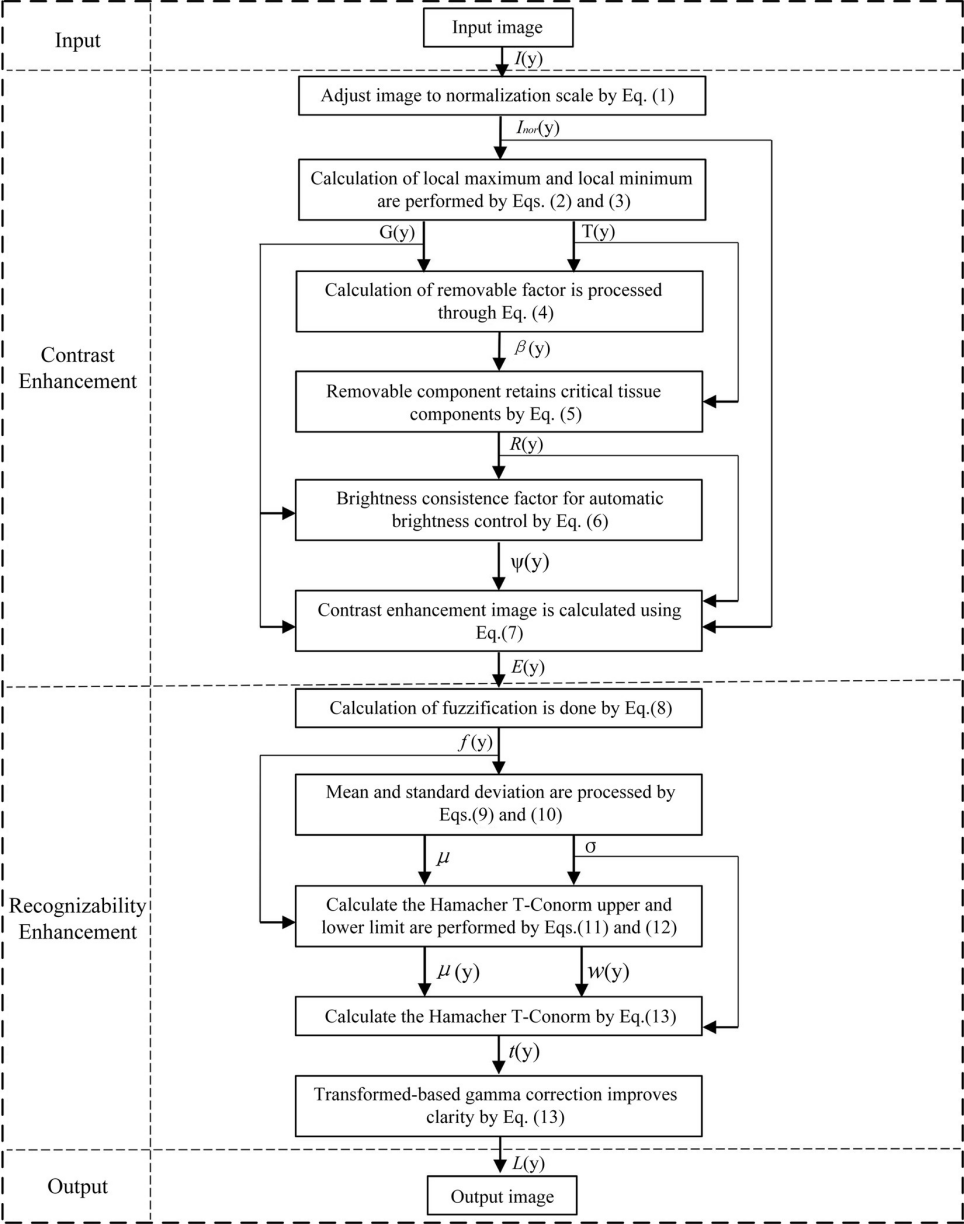
Algorithm flow chart. The input image is processed through stages, yielding an enhanced final output.

We then proposed a method for improving the sharpness of medical images. Finally, we introduced the overall structure of the algorithm, the overall flowchart of proposed method is shown in Figs 2 and 3 shows the algorithm’s flowchart for the proposed method. To summarize, Algorithm 1 outlines the key implementation steps of our ensemble framework for X-ray enhancement.

### Algorithm 1. Pseudocode of the shoulder X-ray image enhancement procedure.

**Algorithm 1** X-ray image enhancement procedure
**Input:** Raw X-ray image *I*(*y*)
**Output:** Enhanced X-ray image *L*(*y*)
**Algorithm**
0: Compute the normalized image *I*_nor_(*y*) using Eq (1)
1: Calculate the local maximum *G*(*y*) and local minimum *T*(*y*) based on Eqs (2) and (3)
2: **For each pixel *p* in *I***_nor_(*y*)
3: Compute the removable factor *β*(*p*) using Eq (4)
4: Calculate removable component *R*(*p*) based on R(p)=β(p)⋅T(p)
5: Adjust brightness consistency using the factor *ψ*(*p*)
6: Generate contrast enhanced image *E*(*p*) by Eq (7)
7: **End**
8: Apply a type-II fuzzy set on *E*(*y*), yielding *f*(*y*) using Eq (8).
9: Calculate mean *μ* and standard deviation σ by Eqs (9) and (10).
10: **For each region** ***r*** **r in** *f*(*y*):
11: Calculate the Hamacher T-Conorm upper *u*(*r*) by Eq (11)
12: Compute lower *w*(*r*) limits by Eq (12)
13: Apply the T-Conorm to enhance sharpness, resulting in *T*(*r*) by Eq (13)
14: Apply gamma correction for clarity, producing *L*(*r*) using Eq (14)
15: **End**
16: Output: Return the enhanced X-ray image *L*(*y*)

### Contrast enhancement module based on tissue attenuation

To resolve the low contrast issue previously mentioned, we propose a method predicated on tissue attenuation, inspired by tissue attenuation techniques [8], that selectively removes specific tissue components, thus retaining the detailed components and ultimately enhancing the image contrast. First, we perform normalization on the input image. The purpose of this process is to standardize the data scale, reduce the dependency on the input range, and improve the model’s ability to generalize new data. The normalized function is defined as:

$$I_{\text{nor}}(y)=\frac{I(y)}{I_{\text{max}}}=D(y)+R(y).$$ (1)

In this context, *y* represents the spatial domain index, uniquely identifying the position of each pixel. *I*_max_ and *I*(*y*) respectively denote the highest grayscale value of the entire image and the grayscale value at a local window in the input image. *D*(*y*) represents the detail component, while *R*(*y*) signifies the removable component. The calculation of local maximum and minimum is based on a windowed range, where *Loy* represents the windowed area determined by the position of pixel y. *G*(*y*) denotes the local maximum, as defined by Eq (2), while the local minimum *T*(*y*) is determined by Eq (3). Notably, *T*(*y*) represents the highest attenuation content within tissues, encompassing both tissue and fat components.


$$G(y)≅\max_{x\in Loy}\;I_{\text{nor}}(y),$$ (2)

$$T(y)≅\min_{x\in Loy}\;I_{\text{nor}}(y).$$ (3)

The removal factor is used to determine the proportion of tissue components to be eliminated. It does not require a manual setting but can be ascertained solely using the local maxima and minima. Local maximum and minimum values from Eqs (2) and (3) are utilized to generate the removable factor *β*(*y*) as Eq (4), where var(*T*(*y*)) is the variance of the local minimum. This approach enables the identification of the most suitable value for the removable factor based on the image, thereby preserving the essential tissue components within the detailed component.


$$\beta (y)=\exp\left(-\frac{G(y)\cdot \text{var}(T(y))}{T(y)}\right).$$ (4)

The removable component *R*(*y*) is jointly determined by the highest attenuation tissue content *T*(*y*) and the removal factor *β*(*y*), with the objective of eliminating non-essential tissue components while retaining critical tissue elements. The calculation formula for the removable component is as follows:

R(y)=β(y)⋅T(y). (5)

Parametric adjustment for brightness consistency which denoted by *ψ*(*y*) [33] is computed by using Eq (6), is utilized for automatic brightness control. Brightness control is a crucial factor in adjusting the visual effects of images during contrast enhancement. It helps maintain the natural appearance of the image while improving the visibility of details and the dynamic range of the image.


$$\psi (y)=\frac{\log\left[1-R(y)\cdot \left(\frac{1}{G(y)}-1\right)\right]}{\log[G(y)]}.$$ (6)

The contrast-enhanced X-ray image *E*(*y*) is calculated using Eq (7), representing the image after contrast enhancement. Here *I*_nor_(*y*) represents the normalized image, while *G*(*y*) and *R*(*y*) denote the local maximum value component and the removal component of the image, respectively. Additionally, *ψ*(*y*) serves as the brightness control parameter.


$$E(y)=\frac{I_{\text{nor}}(y)-R(y)}{G{\left(y\right)}^{\psi (y)}-R(y)}.$$ (7)

### Type-II fuzzy set sharpness enhancement module

To address the issue of insufficient sharpness in X-ray images as discussed earlier, we have employed a sharpness enhancement algorithm based on type-II fuzzy sets, which effectively improves the sharpness of X-ray images. Firstly, we have conducted a fuzzification operation. *F*(*y*) represents the image after normalization, where *E*(*y*) denotes the grayscale value of the pixel determined by the spatial index *y*, max and min respectively represent the maximum and minimum values of the given image. The method for normalization is referenced in Eq (8):

$$f(y)=\frac{E(y)-\min(E(y))}{\max(E(y))-\min(E(y))}.$$ (8)

Then we compute the upper bound denoted by *μ*(*y*) and lower bound denoted by *w*(*y*) of the type-II fuzzy membership function, it is first necessary to calculate the mean *μ* and variance σ of the normalized image, following the formulas provided in Eqs (9) and (10). Inspired by robust optimization method [34] that utilizes mean and variance, we apply similar measures to quantify the distribution of the normalized image. The calculation formulas for the mean and standard deviation are as follows:

$$\mu =\frac{1}{n}\cdot \sum_{i=1}^{n}f_{i},$$ (9)

$$\sigma =\sqrt{\frac{1}{n-1}\cdot \sum_{i=1}^{n}{\left(f_{i}-\mu \right)}^{2}}.$$ (10)

Here, *f*_*i*_ corresponds to the specific positional element in *f*(*y*), and n represents the number of elements *f*_*i*_. The Hamacher T-Conorm upper limit *u*(*y*), based on the gamma correction method proposed by Kallel et al. [35] can be found in Eq (11), where *α* is a hyperparameter used to adjust the level of contrast enhancement, and falls within the range of 0 < *α <* 1. It is particularly noteworthy that when *α* > 0.6, the enhancement of sharpness becomes relatively pronounced.


$$u(y)={\left(f(y)\right)}^{\alpha }+(1-{\left(f(y)\right)}^{\alpha })\cdot {\left(\sigma^{2}\right)}^{\alpha }.$$ (11)

The method for calculating the lower limit is based on the contrast stretching technique from [36] and does not require the introduction of any hyperparameters. The formula for calculating the lower bound is as follows:

$$w(y)=\left(\frac{\alpha \cdot \mu }{\sigma +\alpha }\right)\cdot (f(y)-\alpha \cdot \mu ).$$ (12)

To compute the image enhanced for sharpness, one must first calculate the Hamacher t-conorm, with the method as follows:

$$T(y)=\frac{u(y)+w(y)+\left(\sigma^{2}-2\right)\cdot u(y)\cdot w(y)}{1-(1-\sigma^{2})\cdot u(y)\cdot w(y)}.$$ (13)

Subsequently, *T*(*y*) requires the use of a transformer-based gamma correction method [37] to obtain the enhanced image *L*(*y*), with the formula as below:

$$L(y)=\max(t(y))\cdot {\left(\frac{t(y)}{\max(t(y))}\right)}^{\gamma }.$$ (14)

## Experimental results

In this section, we explore the experiments setting to evaluate the efficacy of our proposed method, including the datasets used, the benchmark methods compared against, and the no-reference image quality assessment metrics utilized. Our method is compared against 10 leading and conventional X-ray image enhancement techniques to illustrate the advantages of our proposed strategy. Moreover, through ablation studies, we validate the effectiveness of our proposed modules. The experimental results revealed that our proposed method shows promising performance in terms of accuracy and efficiency, achieving the best or second-best results in five evaluation metrics. The ablation study further underscores the importance and impact of a sharpness enhancement module based on a type-II fuzzy set in improving image quality, coupled with a contrast enhancement algorithm grounded in tissue attenuation, significantly advances the clarity and detail representation in X-ray

images. Finally, we also conducted generalization tests to validate the effectiveness and robustness of the proposed method on X-ray images of different body parts and across various scenarios.

### Experiments setting

In this section, we meticulously describe the setting of our experiments. Specifically, we conduct a comprehensive validation of our method on the MURA [38] dataset using five no-reference image quality evaluation metrics, comparing it against 10 traditional methods based on X-ray image enhancement. Additionally, we also conducted ablation experiments and generalization tests. Below, we detail the no-reference image dataset MURA used in this study, the comparative methods selected, and the no-reference image quality evaluation metrics employed for assessment.

#### Dataset

The MURA dataset, developed by Stanford University, is an extensive library of musculoskeletal radiographic images, containing 40,561 images manually labeled as normal or abnormal by professional radiologists. This dataset is not only vast in scale, covering a wide range of musculoskeletal conditions, but also supports automated analysis, anomaly detection, and research in medical image processing with its high-quality and diverse data. The public availability of MURA has encouraged participation from researchers worldwide, fostering the development of medical image processing technologies. The decision to use the MURA dataset for X-ray image enhancement research is based on the abundance of precisely annotated images it offers, ensuring data reliability and practicality. Additionally, the variety of musculoskeletal conditions represented in the dataset adds to its diversity, providing a rich resource for research and an ideal platform for evaluating the robustness of our proposed method. On this basis, we randomly selected 12 images from the shoulder subset of the MURA dataset as our experimental data, aiming to deepen the understanding and exploration of image enhancement techniques in the medical field.

#### Compared methods

In our comparative experiments, we utilized the MURA dataset as a benchmark to conduct a comprehensive evaluation of 10 traditional X-ray image enhancement techniques. These selected methods represent a variety of different techniques within the traditional medical image enhancement domain, including CECI [39], CLAHE [40], ECE [41], EGIF [42], FCCE [43], FCE [44], GC [45], HLIPSCS [46], LCA [47], RCEA [48]. The objective of this comparison was to delve into a detailed analysis and assessment of these effective methods in improving the quality of shoulder X-ray images, thereby offering deeper insights and evaluations to the field of medical image processing.

#### No-reference image quality assessment metrics

The quality of shoulder X-ray images primarily depends on factors such as contrast, dynamic range, spatial resolution, noise, and artifacts [49], all of which influence the clarity and diagnostic utility of the images. To effectively evaluate X-ray image enhancement techniques, we utilized multiple no-reference image quality assessment metrics that comprehensively quantify these aspects of visual quality. Among them, the Blind Image Quality Model Evaluator (BIQME) [50] focuses on the naturalness of images, with high scores indicating that the image is closer to a natural visual experience, demonstrating the effectiveness of image processing technologies in maintaining natural colors and textures. The Fog Aware Density Evaluator (FADE) [51] specializes in assessing the clarity and visibility of images, where lower scores signify a low haze density, thus indicating clearer images. The Average Gradient (AG) [52] measures the detail contrast of an image, with higher scores meaning better visibility and detail contrast. Information Entropy (IE) [53] evaluates the richness of information in an image, where an increase in score reflects an increase in image details and information content. The MA [54] metric provides a comprehensive assessment of the visual quality of images produced by the algorithm, with higher scores indicating higher visual quality and better alignment with human visual perception standards. These metrics collectively consider multiple dimensions such as the naturalness, clarity, contrast, and color richness of images, providing a comprehensive set of quantitative evaluation methods for shoulder X-ray image enhancement techniques. This includes both the prominence of image details and the overall visual experience, making the evaluation more objective and comprehensive.

### Qualitative and quantitative comparisons

#### Qualitative comparisons

**Fig 4:**
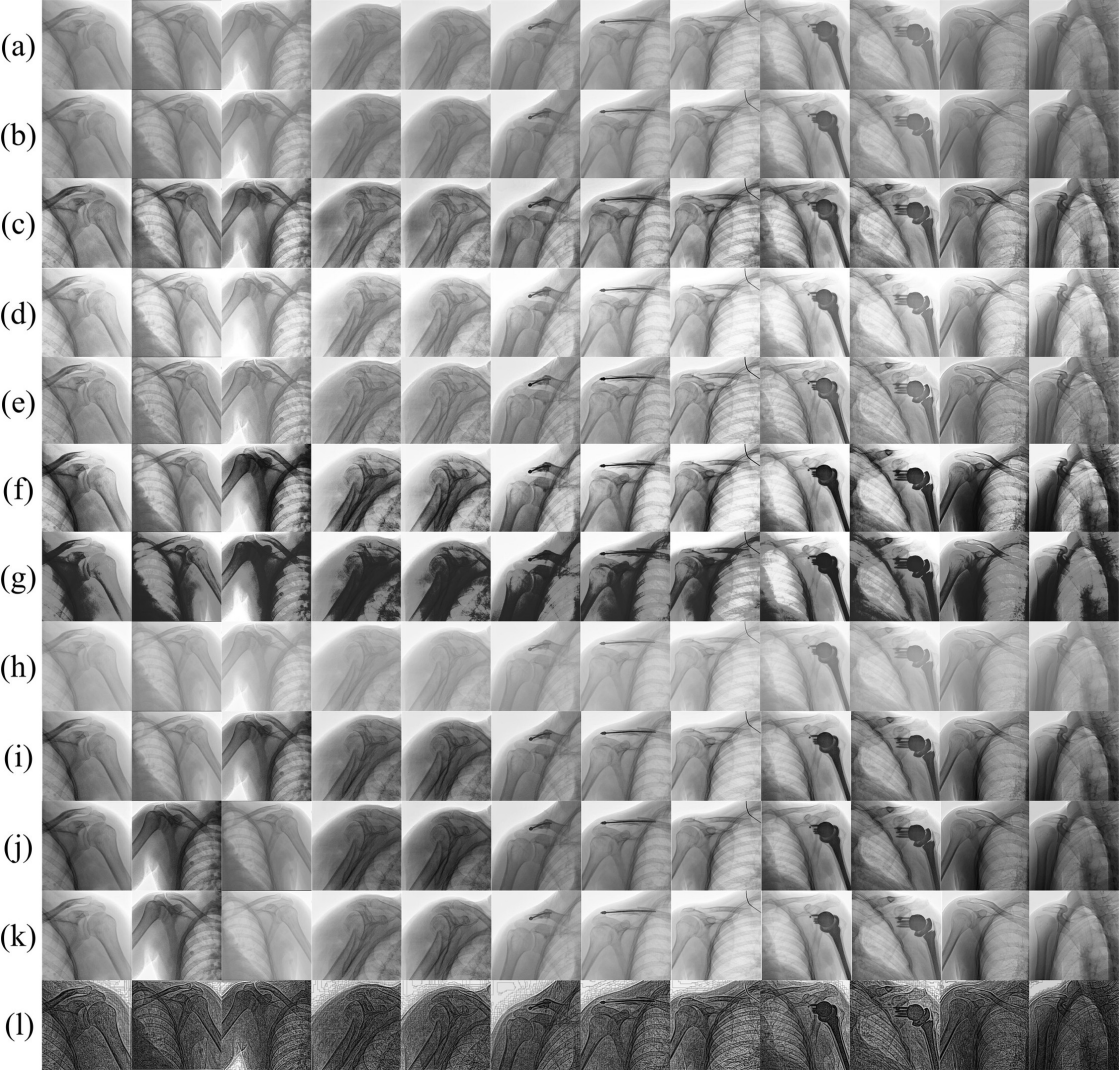
Comparison of X-ray enhancement results between the proposed method and 10 other traditional X-ray enhancement techniques. The images are arranged from left to right, with Image 1 to Image 10 representing the results of the respective traditional enhancement techniques. (a) Input, (b) CECI [39], (c) CLAHE [40], (d) ECE [41], (e) EGIF [42], (f) FCCE [43], (g) FCE [44], (h) GC [45], (i) HLIPSCS [46], (j) LCA [47], (k) RCEA [48], and (l) proposed method.

Fig 4 presents a qualitative comparison of the proposed method with 10 other methods. (a) This line denotes the input data. Turning to the (b) series, the images appear flat in contrast. This lack of contrast may flatten subtle grayscale differences, thereby suppressing detail recognition. This is especially detrimental in cases where differentiation of tissues with close density is required, potentially leading to insufficient diagnosis of certain disease states. In the (c) series, despite a certain enhancement of edges that might benefit the delineation of structures, the effects of over enhancement are evident, leading to non-physiological edges and unnatural texture contrasts, which may mislead diagnosis. In the (d) series, the image processing appears to overly emphasize contrast between light and dark areas, resulting in overexposed or excessively suppressed regions. This not only introduces visual noise but may also mask subtle pathological changes. The black blotches observed in the (f) and (g) series may indicate algorithmic deficiencies in processing low-light or low-signal areas. Information in these regions might be crucial but obscured by incorrect enhancement strategies, affecting the overall readability of the image. The foggy effect in the (h) series further illustrates the complexity of image processing. Such fogging could be due to excessive smoothing by denoising algorithms, thereby reducing image contrast and clarity, making diagnosis more difficult. In the (i) series, the balanced treatment of brightness and contrast might aim to reduce the previously mentioned problems, yet there are still areas of blurring due to over-processing, which is unacceptable in diagnostic situations requiring precise measurements. As for the (j) series, while maintaining a richness of detail, managing brightness and darkness is extremely important. Overly bright areas in the image may lead to information loss, while overly dark areas may conceal potential pathological features. Finally, the enhancement techniques displayed in these X-ray images, as shown in (k), may not have adequately balanced the enhancement of contrast with the preservation of details, resulting in over-smoothing or increased noise in some images. Taking the (l) series as an example, we can observe that the algorithm has enhanced contrast and sharpness, improving the visualization of anatomical structures, particularly at the boundaries between bone and soft tissue, while avoiding degradation in image quality such as foggy blurring or artifact generation. This clarity enhancement has been achieved by optimizing image contrast and sharpness while preserving the natural textures and shadows of the tissues, which is vital for identifying potential subtle pathologies. Finally, the initial feedback from the radiologist indicates that the method significantly enhances the visibility of fine structures and anatomical details. The improvement in contrast and edge delineation, particularly in the lung and rib areas, contributes to more accurate diagnosis of subtle abnormalities.

#### Quantitative comparisons

In our comprehensive evaluation of the effects on X-ray image enhancement, contrast optimization, and noise reduction, a series of quantitative metrics were employed for analysis. Specifically, by applying BIQME, FADE, AG, IE, and MA as five evaluation standards on the MURA dataset, we compared the performance of 10 different image processing methods. The analysis revealed that our proposed strategy demonstrated promising results, achieving the top performance in four out of five evaluation metrics and securing second place in the remaining metric on the selected dataset. Further validation on a larger dataset is necessary to confirm its overall effectiveness. This performance emphatically demonstrates the superior capabilities of our method in enhancing image quality, adjusting contrast, and reducing noise. In comparative experiments, the performance of our proposed method against the other 10 methods on the BIQME metric, as shown in Tables 3 and 8, indicates that our method ranked best based on the scores of eight pictures recorded and obtained the best overall score. Moreover, in the assessment of the FADE metric, our method achieved the best results across all twelve images shown in Table 4 and ranked first in the overall average score in Table 8. Under the AG metric, our approach also performed best for the 12 images displayed in Table 5 and led the overall average scores in Table 8. In the IE metric evaluation, our method performed well, securing the best scores for three out of twelve evaluated images, the remaining nine images are either second in ranking or very close to it in Table 6 and ranking second in the overall score summary of Table 8. In the assessment of the MA metric, our algorithm demonstrated exceptional performance, achieving the best or second-best results in 10 evaluated images in Table 7, and ranking first in the overall score summary of Table 8. Additionally, Table 9 presents the computational complexity analysis, comparing the execution times of various algorithms. The proposed method, while delivering enhanced shoulder X-ray image quality, has a relatively higher computational cost, which may limit its real-time applicability. Future work will focus on optimizing the algorithm to improve efficiency without compromising enhancement performance.

**Table 3:** Comparative assessment of BIQME scores across diverse methods for distinct images. The best result is bold, and the second-best result is underlined.

Method/Image	CECI [39]	CLAHE [40]	ECE [41]	EGIF [42]	FCCE [43]	FCE [44]	GC [45]	HLIPSCS [46]	LCA [47]	RCEA [48]	proposed method
Image 1	0.3629	0.4186	0.3658	0.3712	0.5162	0.5397	0.3141	0.4484	0.4740	0.4031	**0.5714**
Image 2	0.3834	0.4772	0.3953	0.4142	0.5950	**0.6000**	0.3394	0.5021	0.5385	0.4480	0.5979
Image 3	0.3697	0.4606	0.3983	0.4015	**0.6044**	0.5284	0.3199	0.4986	0.5044	0.4488	0.5613
Image 4	0.4147	0.5240	0.4299	0.4206	**0.6098**	0.5101	0.3469	0.5425	0.5370	0.4711	0.4972
Image 5	0.3919	0.4794	0.4073	0.4022	0.4472	**0.5641**	0.3540	0.4031	0.3983	0.3611	0.5575
Image 6	0.3407	0.4729	0.3556	0.3561	0.5915	0.5510	0.2924	0.5146	0.5476	0.4618	**0.5927**
Image 7	0.3421	0.3947	0.3566	0.3477	0.5136	0.5505	0.2981	0.4476	0.4833	0.3961	**0.5665**
Image 8	0.3439	0.3989	0.3561	0.3516	0.5171	0.5482	0.3017	0.4903	0.5249	0.4316	**0.5893**
Image 9	0.3796	0.4516	0.3905	0.4007	0.4977	0.5514	0.3285	0.4108	0.4231	0.3793	**0.5805**
Image 10	0.3660	0.4529	0.3782	0.4007	0.4824	0.5639	0.3170	0.4049	0.4148	0.3658	**0.5806**
Image 11	0.3796	0.4706	0.3732	0.4235	0.4957	0.5745	0.3309	0.4134	0.4308	0.3737	**0.5853**
Image 12	0.4225	0.5132	0.4124	0.4503	0.5506	0.5449	0.3726	0.5181	0.5347	0.4669	**0.5827**

**Table 4:** Comparative assessment of FADE scores across diverse methods for distinct images. The best result is bold, and the second-best result is underlined.

Method/Image	CECI [39]	CLAHE [40]	ECE [41]	EGIF [42]	FCCE [43]	FCE [44]	GC [45]	HLIPSCS [46]	LCA [47]	RCEA [48]	proposed method
Image 1	3.1252	1.5065	2.4686	1.9022	1.4764	1.5721	4.0258	1.9820	2.0205	2.5245	**0.2648**
Image 2	2.5940	1.2441	2.1677	1.5153	1.0283	1.2042	3.3741	1.3930	1.2155	1.6787	**0.2249**
Image 3	2.4273	1.1853	1.9162	1.4108	0.8812	1.2738	3.1698	1.3720	1.4006	1.8209	**0.2003**
Image 4	2.3656	1.2131	1.7788	1.4865	0.9587	1.3060	3.0504	1.3559	1.4502	1.9734	**0.2296**
Image 5	2.6100	1.3823	2.1248	1.6797	1.8448	1.4355	3.4656	2.4017	2.4315	2.9157	**0.2258**
Image 6	2.6998	1.2565	2.1801	1.5698	0.9209	1.3567	3.4052	1.3143	1.1555	1.5467	**0.2101**
Image 7	3.2725	1.6402	2.5767	1.9731	1.3024	1.5811	3.9814	1.8277	1.7828	2.2890	**0.2739**
Image 8	3.5575	1.7875	2.7888	2.2235	1.3838	1.6788	4.2102	1.8345	1.6487	2.1571	**0.3072**
Image 9	3.3859	1.8908	2.4960	2.2214	1.8263	1.6866	4.1177	2.5926	2.7812	3.0678	**0.3677**
Image 10	2.9957	1.5764	2.4274	1.6693	1.6975	1.5385	3.8448	2.4497	2.3688	2.9487	**0.2781**
Image 11	2.5268	1.3284	2.3400	1.5284	1.4673	1.3450	3.2984	2.1775	2.0392	2.5251	**0.2756**
Image 12	2.4551	1.2940	2.3294	1.5026	1.3533	1.5171	3.3225	1.6777	1.6050	2.0718	**0.2529**

**Table 5:** Comparative assessment of AG scores across diverse methods for distinct images. The best result is bold, and the second-best result is underlined.

Method/Image	CECI [39]	CLAHE [40]	ECE [41]	EGIF [42]	FCCE [43]	FCE [44]	GC [45]	HLIPSCS [46]	LCA [47]	RCEA [48]	proposed method
Image 1	1.9337	4.2410	2.9503	3.5970	4.4912	3.5597	1.5797	2.9923	2.4000	2.5379	**18.6889**
Image 2	2.4983	5.3289	3.6717	4.7221	6.0619	5.1823	2.0121	4.2306	4.3617	3.8734	**22.1364**
Image 3	2.6693	5.9069	4.4689	5.1484	7.5465	5.1225	2.2853	4.4081	3.5332	3.6068	**21.1281**
Image 4	2.3834	5.1653	4.0552	4.4964	5.7562	4.0924	2.0384	3.4680	2.7336	3.0387	**15.9013**
Image 5	2.3229	4.5045	3.4904	3.9347	3.6500	3.9122	1.9454	2.5579	2.3820	2.3462	**18.8979**
Image 6	2.5792	5.5777	3.6991	4.8800	6.3217	4.9850	2.0599	4.6455	4.7544	4.3747	**25.0120**
Image 7	2.0018	4.5499	3.2981	3.7634	5.6225	4.1441	1.6795	3.4743	3.1642	3.1131	**18.7068**
Image 8	1.8390	4.1662	3.0167	3.4373	5.4229	3.9499	1.5378	3.3552	3.3000	3.2240	**18.4905**
Image 9	1.8830	3.9729	2.9776	3.4349	4.4878	3.5298	1.5234	2.4318	2.0937	1.9936	**15.9869**
Image 10	2.1463	4.5117	3.1627	3.9920	4.7157	4.0963	1.7365	2.6534	2.4654	2.3042	**20.4107**
Image 11	2.7564	5.5624	3.3593	5.1812	5.2372	5.1221	2.1925	3.2506	3.1709	2.8763	**22.0103**
Image 12	2.4739	4.8886	3.0746	4.5628	4.4159	4.4908	1.9831	3.4459	3.0307	3.0788	**20.3262**

**Table 6:** Comparative assessment of IE scores across diverse methods for distinct images. The best result is bold, and the second-best result is underlined.

Method/Image	CECI [39]	CLAHE [40]	ECE [41]	EGIF [42]	FCCE [43]	FCE [44]	GC [45]	HLIPSCS [46]	LCA [47]	RCEA [48]	proposed method
Image 1	6.9461	7.2739	7.0003	7.0634	**7.7064**	7.0290	6.5421	7.4026	7.3493	7.2213	7.5002
Image 2	6.8834	7.5280	6.9520	7.1356	**7.7803**	7.1787	6.4724	7.5085	7.4967	7.3767	7.5914
Image 3	6.8452	7.3583	7.2663	7.0262	**7.8545**	7.0962	6.5395	7.4070	7.2951	7.2503	7.2868
Image 4	6.8825	7.4966	7.3454	6.9881	**7.6800**	6.8501	6.5776	7.2995	7.1251	7.2060	7.0745
Image 5	6.7924	7.4295	7.1549	6.9368	**7.4388**	7.0346	6.4194	6.9488	6.8387	6.5656	7.3251
Image 6	6.8130	7.5743	6.9164	6.9525	**7.8336**	6.9598	6.4195	7.5544	7.5217	7.4673	7.5030
Image 7	6.7517	7.2226	6.9381	6.9047	**7.6279**	6.8912	6.3687	7.2932	7.2272	7.1592	7.4889
Image 8	6.7199	7.2172	6.8636	6.8593	**7.6419**	6.8574	6.3180	7.3805	7.3207	7.2808	7.5649
Image 9	6.7227	7.3795	6.7391	6.8844	7.3866	6.6609	6.2905	6.9649	6.9021	6.6867	**7.4838**
Image 10	6.7794	7.4258	6.8009	6.9335	7.4490	7.0277	6.3527	7.0215	7.0250	6.7534	**7.5647**
Image 11	7.0445	7.4667	6.7818	7.1732	7.4857	7.3083	6.5832	7.1807	7.2818	6.9361	**7.6583**
Image 12	7.1712	7.6260	7.0658	7.2974	**7.6797**	7.2263	6.7456	7.5107	7.5446	7.3411	7.5858

**Table 7:** Comparative assessment of MA scores across diverse methods for distinct images. The best result is bold, and the second-best result is underlined.

Method/Image	CECI [39]	CLAHE [40]	ECE [41]	EGIF [42]	FCCE [43]	FCE [44]	GC [45]	HLIPSCS [46]	LCA [47]	RCEA [48]	proposed method
Image 1	3.2100	6.6481	3.6825	5.2295	7.3559	7.2379	3.1359	3.7079	3.3072	3.2953	**8.5751**
Image 2	3.8676	7.3583	5.9394	7.1165	8.0336	8.3029	3.4621	6.4553	6.5270	6.0055	**8.6635**
Image 3	3.8989	8.6630	7.0831	8.4635	**8.8101**	8.5786	3.4606	7.4287	6.2237	6.2410	8.6754
Image 4	3.5656	8.4726	6.8714	8.2771	**8.7978**	8.3561	3.3352	6.4746	4.9850	4.7046	8.5849
Image 5	3.2434	6.4166	4.4548	5.2307	5.1699	7.2410	3.2271	3.2818	3.2553	3.2283	**8.5612**
Image 6	3.7218	8.1226	6.1902	7.5721	**8.8880**	8.6948	3.3343	7.1465	7.3226	6.7742	8.5422
Image 7	3.2380	6.3084	4.9691	5.3473	8.1063	7.9638	3.2028	5.1353	4.3193	4.6605	**8.5133**
Image 8	3.2155	7.3061	5.1576	5.9668	8.5078	**8.5079**	3.2522	6.3362	5.7700	5.7873	8.4659
Image 9	3.3040	5.7191	4.3759	5.2007	7.5390	7.2112	3.2501	3.4176	3.3601	3.3730	**8.4727**
Image 10	3.5183	7.2854	5.5516	6.7230	7.9706	8.4442	3.4090	4.5433	4.1556	3.8114	**8.6955**
Image 11	4.2444	7.9589	5.9839	7.8740	8.0211	8.0898	3.6263	5.7807	5.4820	4.6781	**8.7104**
Image 12	3.7993	7.5305	4.6217	7.5795	7.5893	7.9480	3.4639	5.4939	4.3205	4.8002	**8.7156**

**Table 8:** Comparison of average metric value between different methods. The best result is bold, and the second-best result is underlined.

Metric\Method	CECI [39]	CLAHE [40]	ECE [41]	ECLF [42]	FCCE [43]	FCE [44]	GC [45]	HLIPSCS [46]	LCA [47]	RCEA [48]	proposed method
BIQME↑ [50]	0.3748	0.4596	0.3849	0.3950	0.5351	0.5522	0.3263	0.4662	0.4843	0.4173	**0.5719**
FADE ↓ [51]	2.8346	1.4421	2.2995	1.7236	1.3451	1.4580	3.6055	1.8649	1.8250	2.2933	**0.2592**
AG↑ [52]	2.2906	4.8647	3.4354	4.2625	5.3108	4.3489	1.8812	3.4095	3.1158	3.0306	**19.8080**
IE↑ [53]	6.8627	7.4165	6.9854	7.0129	**7.6304**	7.0100	6.4691	7.2894	7.2440	7.1037	7.4690
Ma↑ [54]	3.5689	7.3158	5.4068	6.7151	7.8991	8.0480	3.3466	5.4335	4.9190	4.7800	**8.5980**

**Table 9:** Comparison of execution time for different X-ray image enhancement methods.

Methods	Environment	Execution Time (s)
CECI [39]	MATLAB/CPU	0.2609
CLAHE [40]	MATLAB/CPU	1.5305
ECE [41]	MATLAB/CPU	0.7238
EGIF [42]	MATLAB/CPU	0.6766
FCCE [43]	MATLAB/CPU	0.0289
FCE [44]	MATLAB/CPU	0.4982
GC [45]	MATLAB/CPU	0.1734
HLIPSCS [46]	MATLAB/CPU	0.5858
LCA [47]	MATLAB/CPU	0.3599
RCEA [48]	MATLAB/CPU	0.0269
Proposed method	MATLAB/CPU	1.8287

### Ablation study

This section provides a detailed analysis of the effectiveness of the contrast and sharpness enhancement modules in our proposed algorithm on the MURA dataset through an ablation study. By removing the sharpness enhancement module, we assess its overall impact on the approach, effectively illustrating the significant role of the sharpness enhancement module in enhancing algorithm performance. By also removing the contrast enhancement module, we further dissect intricacies of the system, revealing the integral part this module plays alongside sharpness enhancement in boosting the algorithm’s efficacy.

**Fig 5:**
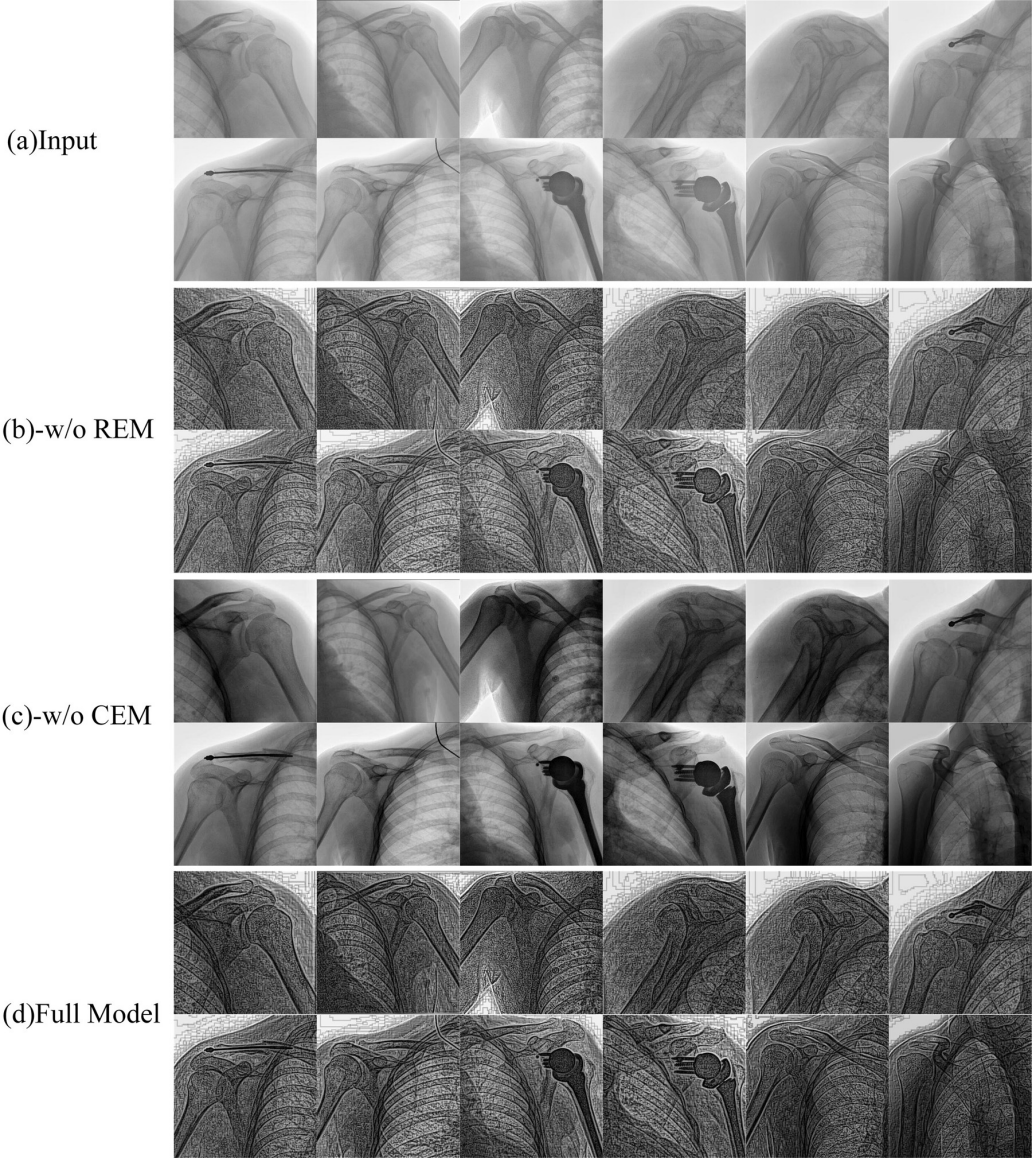
Comparative visualization reveals: (a) input low-contrast images; (b) -w/o REM results with enhanced contrast but no sharpness improvement; (c) -w/o CEM outcomes with unclear tissue differentiation; (d) Full Model enhancements showing marked sharpness and clarity improvements.

Firstly, a qualitative comparative experiment was conducted. In Fig 5, "(a) Input" refers to the original image, characterized by its relatively blurry effect and low contrast, where key skeletal and tissue sections are not sufficiently highlighted, potentially limiting the effectiveness of precise image analysis. "(b) -w/o REM" represents the method after removing the sharpness enhancement module, showing the enhancement results post-removal, where the contrast and detail of images are significantly improved compared to the original, making bone information and key tissues clearer. Despite this, compared to the full model, this type of enhancement might still fall short in terms of overall image sharpness. "(c) -w/o CEM" illustrates the method following the removal of the contrast enhancement module. Compared to the full model, it is visually apparent that images generated without CEM lack the clarity needed to distinguish between bones and tissues effectively. "(d) Full Model" represents our proposed complete method, where we can observe an all-encompassing enhancement effect; not only are the details of bones and key tissues more pronounced, but the overall sharpness and clarity of the image are also significantly improved, greatly enhancing the observer’s ability to discern details. This is especially critical when conducting a detailed interpretation of images. Table 10 meticulously presents the results of an ablation experiment conducted on the MURA dataset, where key modules were removed to compare the performance of various model configurations using five non-reference evaluation metrics, aiming to precisely reveal the specific contributions of each independent module to the overall model performance. Specifically, "-w/o REM" demonstrates the performance of model without the sharpness enhancement module, while "-w/o CEM" indicates the effects after the removal of the contrast enhancement module. It is evident from Table 10 that the presence of sharpness and contrast enhancement modules is crucial for improving model performance. For instance, the full model scores the lowest on the FADE metric, signifying its superior performance in enhancing image quality. At the same time, BIQME, AG, IE, and Ma metrics all score the highest in the full model, further validating the importance of each module in performance enhancement. Moreover, the significantly high score on the AG metric particularly highlights the superior performance of the full model compared to the ablated models lacking any key module, underscoring the profound impact of the synergistic work between modules in the model, especially in terms of enhancing image clarity and contrast.

**Table 10:** The results of the ablation experiments of the different modules.

Ablated Model	BIQME↑ [50]	FADE↓ [51]	AG↑ [52]	IE↑ [53]	Ma↑ [54]
-w/o REM	0.5386	0.3784	18.8843	7.4215	8.5837
-w/o CEM	0.5289	1.2735	3.5630	7.3710	5.7433
Full Model	**0.57191**	**0.2592**	**19.8080**	**7.4690**	**8.5980**

### Generalization test

**Fig 6:**
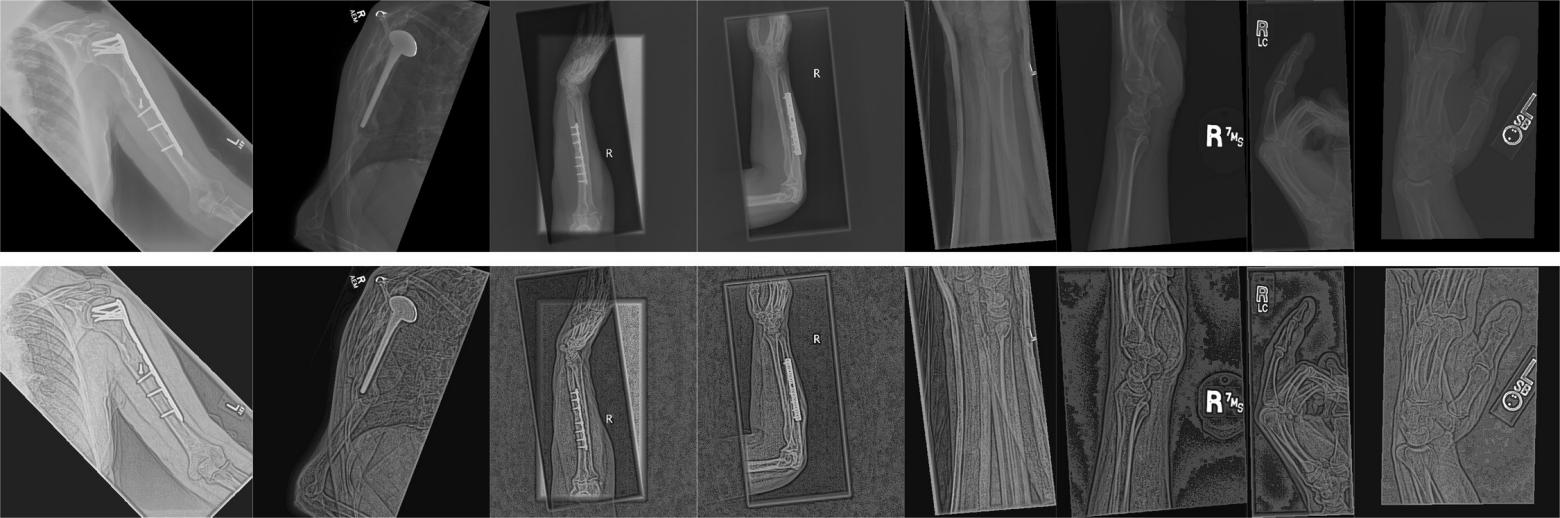
Generalization test of the proposed method on X-ray images of various body parts, including the humerus, forearm, wrist, and fingers, taken from the MURA dataset.

The generalization test for the shoulder X-ray image enhancement algorithm focuses on thoroughly evaluating its robustness and ability to generalize across various types of X-ray images, extending beyond shoulder X-rays to images of other anatomical structures. This is critical for assessing the algorithm’s applicability and reliability in real-world clinical scenarios, where X-ray images of different body parts are frequently analyzed. To conduct this evaluation, the proposed method was applied to a broader range of X-ray images by randomly sampling from the MURA dataset, specifically selecting images of the humerus, forearm, wrist, and fingers. These images were then processed using the proposed enhancement technique to test its effectiveness across diverse anatomical regions. As depicted in Fig 6, the top row presents the original X-ray images, while the bottom row displays the enhanced images produced by the algorithm. The results clearly demonstrate that the proposed method significantly improves the visibility of critical bone structures and effectively enhances image contrast and sharpness, even in complex anatomical regions. This showcases the method’s potential for widespread clinical utility, providing reliable and consistent enhancement across various X-ray imaging scenarios, thus improving diagnostic accuracy and aiding medical professionals in more precise interpretations.

## Discussion

In this study, we proposed a novel enhancement method for shoulder X-ray images, improving both sharpness and contrast by integrating tissue attenuation techniques with a type-II fuzzy set-based algorithm. Our method significantly enhances image quality, particularly in low-contrast and low-sharpness images, preserving details and highlighting critical features, which improves diagnostic and clinical utility. When compared to 10 traditional enhancement methods using the partial MURA dataset, our method showed superior results, especially in challenging shoulder X-ray scenarios. It enhances contrast and sharpness, making bone structures clearer and aiding in the detection of potential lesions.

However, the method has limitations. It may not perform well on extremely blurry or noisy images, and its computational cost increases with larger datasets, limiting the scalability for real-time or large-scale applications. Additionally, the algorithm’s performance is sensitive to parameter settings, requiring fine-tuning for optimal results. Automating parameter selection or developing adaptive methods would be crucial for consistent performance across various imaging scenarios.

## Conclusion

In this study, we proposed a comprehensive enhancement framework for shoulder X-ray images, which improves image contrast through tissue attenuation and enhances sharpness using a type-II fuzzy set-based approach. We tested the method on several shoulder X-ray images from the MURA dataset, comparing it with 10 traditional enhancement techniques, achieving the best results. Additionally, the generalization tests confirmed its effectiveness for X-ray images of other body parts, highlighting its broad applicability. This method improves shoulder X-ray image quality, particularly in low-contrast and detail-lacking images, aiding radiologists in more accurate analyses and better diagnostic accuracy. Future work could focus on optimizing the algorithm for improved efficiency and exploring its potential in more complex medical imaging scenarios. Our research aims to enhance diagnostic accuracy, reduce follow-up rates, and accelerate clinical decision-making.

## Supporting information

S1 Dataset Partial MURA dataset for experimental evaluation. (ZIP)

S1 Appendix (DOCX)
