# Supplementary figures and images for: Adaptive enhancement of shoulder x-ray images using tissue attenuation and type-II fuzzy sets
Source: PLoS One. 2025 Feb 6;20(2):e0316585. doi: 10.1371/journal.pone.0316585 (PMC11801559; doi:10.1371/journal.pone.0316585)

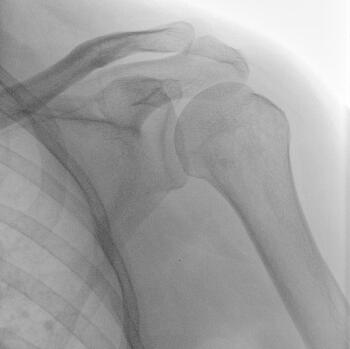

Supplement: S1 Dataset — (ZIP) [file pone.0316585.s001.zip › dataset/1.jpg]

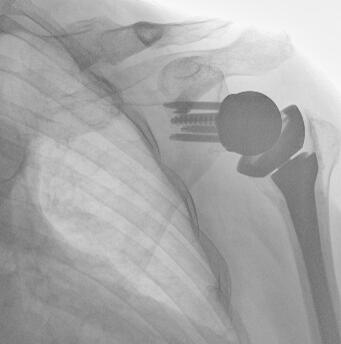

Supplement: S1 Dataset — (ZIP) [file pone.0316585.s001.zip › dataset/10.jpg]

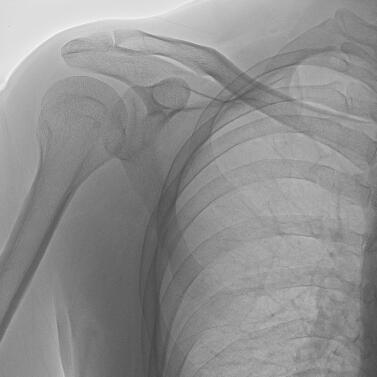

Supplement: S1 Dataset — (ZIP) [file pone.0316585.s001.zip › dataset/11.jpg]

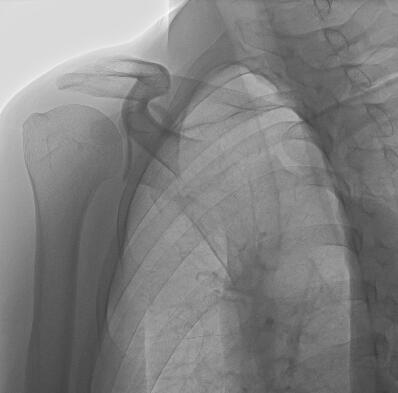

Supplement: S1 Dataset — (ZIP) [file pone.0316585.s001.zip › dataset/12.jpg]

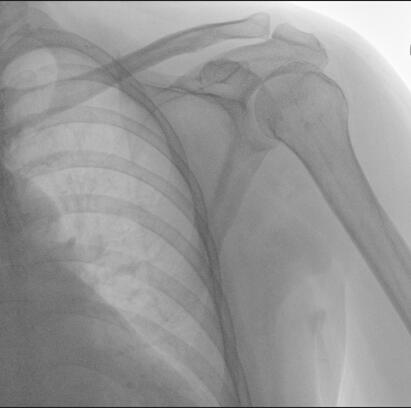

Supplement: S1 Dataset — (ZIP) [file pone.0316585.s001.zip › dataset/2.jpg]

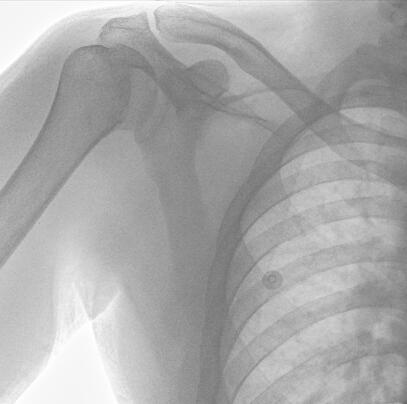

Supplement: S1 Dataset — (ZIP) [file pone.0316585.s001.zip › dataset/3.jpg]

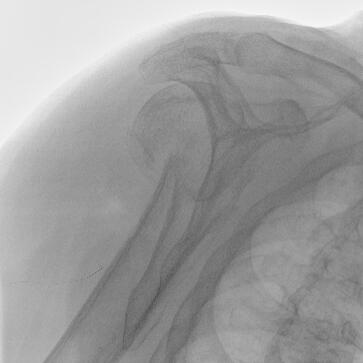

Supplement: S1 Dataset — (ZIP) [file pone.0316585.s001.zip › dataset/4.jpg]

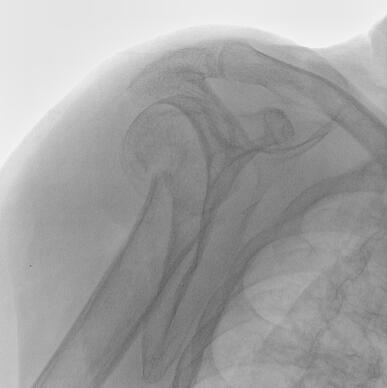

Supplement: S1 Dataset — (ZIP) [file pone.0316585.s001.zip › dataset/5.jpg]

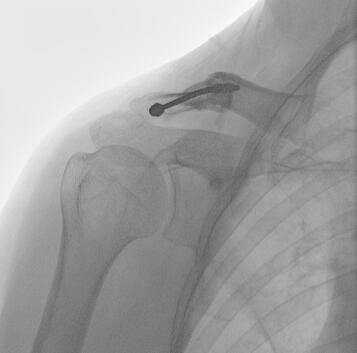

Supplement: S1 Dataset — (ZIP) [file pone.0316585.s001.zip › dataset/6.jpg]

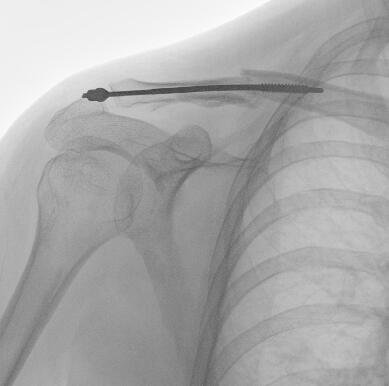

Supplement: S1 Dataset — (ZIP) [file pone.0316585.s001.zip › dataset/7.jpg]

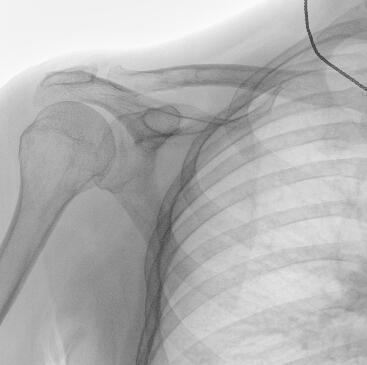

Supplement: S1 Dataset — (ZIP) [file pone.0316585.s001.zip › dataset/8.jpg]

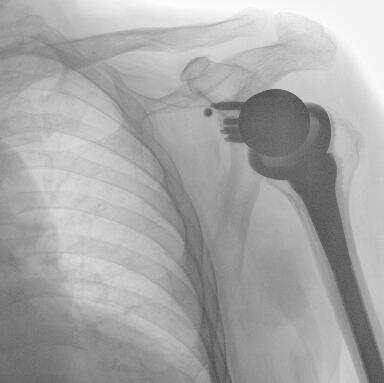

Supplement: S1 Dataset — (ZIP) [file pone.0316585.s001.zip › dataset/9.jpg]
