# Supplementary material for: Adaptive enhancement of shoulder x-ray images using tissue attenuation and type-II fuzzy sets
Source: PLoS One. 2025 Feb 6;20(2):e0316585. doi: 10.1371/journal.pone.0316585 (PMC11801559; doi:10.1371/journal.pone.0316585)
Supplement: S1 Appendix — (DOCX) [file pone.0316585.s002.docx]

**Appendix**

**Table A1 Definitions of Abbreviations and Their Corresponding Contexts**

| Abbreviation | Meaning/Context |
| --- | --- |
| GAN | Generative Adversarial Network |
| CNN | Convolutional Neural Network |
| MRI | Magnetic Resonance Imaging |
| BIQME | Blind Image Quality Model Evaluator |
| FADE | Fog Aware Density Evaluator |
| AG | Average Gradient |
| IE | Information Entropy |
| MA | Ma's Assessment Metric |
| CECI | Contextual Enhancement Contrast Improvement |
| CLAHE | Contrast-Limited Adaptive Histogram Equalization |
| ECE | Efficient contrast enhancement |
| EGIF | Effective Guided Image Filtering |
| FCCE | Fuzzy-Contextual Contrast Enhancement |
| FCE | Fuzzified Contrast Enhancement |
| GC | Gamma Correction |
| HLIPSCS | High-Level Image Processing with Contrast Stretching |
| RCEA | Rapid Contrast Enhancement Algorithm |
| CNN | Convolutional Neural Network |
| DICOM | Digital Imaging and Communications in Medicine |
